# A multicentre phase II study of cisplatin and gemcitabine for malignant mesothelioma

**DOI:** 10.1038/sj.bjc.6600505

**Published:** 2002-08-27

**Authors:** A K Nowak, M J Byrne, R Williamson, G Ryan, A Segal, D Fielding, P Mitchell, A W Musk, B W S Robinson

**Affiliations:** Department of Medical Oncology, Sir Charles Gairdner Hospital, Verdun Street, Nedlands, WA 6009 Australia; Department of Respiratory Medicine, Sir Charles Gairdner Hospital, Verdun Street, Nedlands, WA 6009 Australia; Department of Medicine, University of Western Australia, Verdun Street, Nedlands, WA 6009 Australia; Department of Public Health, University of Western Australia, Nedlands, WA 6009 Australia; Pathcentre, Sir Charles Gairdner Hospital, Verdun Street, Nedlands, WA 6009 Australia; Department of Respiratory Medicine, Princess Alexandra Hospital, Brisbane, Australia; Department of Medical Oncology, Austin & Repatriation Medical Centre, Melbourne, Australia

**Keywords:** malignant mesothelioma, gemcitabine, cisplatin, quality of life, drug treatment

## Abstract

Our previous phase II study of cisplatin and gemcitabine in malignant mesothelioma showed a 47.6% (95% CI 26.2–69.0%) response rate with symptom improvement in responding patients. Here we confirm these findings in a multicentre setting, and assess the effect of this treatment on quality of life and pulmonary function. Fifty-three patients with pleural malignant mesothelioma received cisplatin 100 mg m^−2^ i.v. day 1 and gemcitabine 1000 mg m^−2^ i.v. days 1, 8, and 15 of a 28 day cycle for a maximum of six cycles. Quality of life and pulmonary function were assessed at each cycle. The best response achieved in 52 assessable patients was: partial response, 17 (33%, 95% CI 20–46%); stable disease, 31 (60%); and progressive disease, four (8%). The median time to disease progression was 6.4 months, median survival from start of treatment 11.2 months, and median survival from diagnosis 17.3 months. Vital capacity and global quality of life remained stable in all patients and improved significantly in responding patients. Major toxicities were haematological, limiting the mean relative dose intensity of gemcitabine to 75%. This schedule of cisplatin and gemcitabine is active in malignant mesothelioma in a multicentre setting. Investigation of alternative scheduling is needed to decrease haematological toxicity and increase the relative dose intensity of gemcitabine whilst maintaining response rate and quality of life.

*British Journal of Cancer* (2002) **87**, 491–496. doi:10.1038/sj.bjc.6600505 www.bjcancer.com

© 2002 Cancer Research UK

## 

Malignant mesothelioma is an aggressive incurable tumour that is increasing in incidence in a number of countries (de Klerk *et al*, 1989; Peto *et al*, 1995). The median survival from diagnosis ranges from 6 to 18 months (Rusch *et al*, 1995) and conventional treatments have not improved this poor prognosis (Ong and Vogelzang, 1996; Sterman *et al*, 1999). We have previously reported a single institution phase II study of the use of cisplatin and gemcitabine in the treatment of pleural malignant mesothelioma. Ten out of 21 patients (47.6%) achieved a partial response of their disease (95% CI 26.2–69.0%) (Byrne *et al*, 1999). The median duration of response was 5.8 months, progression free survival 5.8 months, and median survival from start of treatment 9.4 months. The median survival of patients from the date of diagnosis was 12.9 months. Although formal quality of life assessments were not performed, nine of the 10 responding patients had significant improvement in symptoms. Serial measurements of vital capacity were performed on three of the responders, all showed a significant increase during the period of remission.

Following these encouraging results, we designed this multicentre phase II study to examine the objective response rate and survival of patients treated with the same dose and schedule of cisplatin and gemcitabine in a multicentre setting. We also measured quality of life and lung function to assess the clinical benefit of this palliative therapy.

## PATIENTS AND METHODS

### Eligibility criteria

Patients were accrued from six tertiary referral centres across Australia. Eligible patients had a histologically or cytologically confirmed diagnosis of malignant pleural mesothelioma, age ⩽75 years, ECOG performance status of 0–2, and a life expectancy of more than 12 weeks. All patients had had either bidimensionally measurable disease of 1.5×1.5 cm or greater, or unidimensionally measurable disease consisting of pleural tumour of at least 1.5 cm in thickness on thoracic CT scan. All had adequate haematological parameters (total leucocyte count >3.0×10^9^ l^−1^; granulocyte count >1.5×10^9 ^l^−1^; platelet count >100×10^9^ l^−1^; and haemoglobin >10 g dl^−1^), adequate renal function (serum creatinine <120 μmol l^−1^), and adequate hepatic function (total bilirubin <1.5×upper limit of normal (ULN); alanine transaminase and alkaline phosphatase <3×ULN). Patients were ineligible if they had had prior systemic chemotherapy for malignant mesothelioma, radiotherapy to all measurable lesions, or a second primary malignancy diagnosed within the past 10 years. Pregnant or lactating women and patients with other serious medical disorders incompatible with the study were also considered ineligible. Patients found to have non-measurable disease on central radiology review were not considered eligible for assessment of response. The protocol was approved by the Committee for Human Rights of the University of Western Australia and the Sir Charles Gairdner Hospital Clinical Drug Trials Committee, and also by the ethics committee of each participating centre. Written informed consent was obtained from each patient prior to study entry.

### Treatment administration

All patients received cisplatin 100 mg m^−2^ intravenously over 1 h on day 1 and gemcitabine 1000 mg m^−2^ over 30 min on days 1, 8, and 15. On day 1, cisplatin was administered before gemcitabine. Cisplatin was administered as an inpatient or outpatient according to individual institutional practice but with a minimum of 3 l intravenous hydration and magnesium supplementation. Prophylactic antiemetic therapy with 5-hydroxytryptamine (5-HT_3_) antagonists and dexamethasone was given intravenously on days 1, 8 and 15. Oral 5-HT_3_ antagonists and/or dexamethasone and/or phenothiazine antiemetics orally or by suppository were continued for 3–5 days following cisplatin.

Cycles were repeated every 28 days. In the absence of disease progression or unacceptable toxicity, patients were scheduled to receive six cycles. A complete blood count and differential, serum electrolytes, creatinine, bilirubin, ALT, alkaline phosphatase, albumin and total protein were performed on day 1 of each cycle. On days 8 and 15, a full blood count and differential were performed.

### Dose adjustments

No dose escalations were permitted. On days 1, 8 and 15, the dose of gemcitabine was adjusted according to the level of myelosuppression on that day. The gemcitabine dose was reduced to 50% if the total leucocyte count was 2.0–2.9×10^9^ l^−1^, and omitted (or delayed 1 week for day 1) at <2.0×10^9^ l^−1^. Gemcitabine was reduced to 50% if the platelet count was 50–74×10^9^ l^−1^, and omitted at <50×10^9^ l^−1^. Cisplatin was not adjusted for myelosuppression. Treatment with growth factors (G-CSF) was not allowed unless there was evidence of severe myelosuppression. On days 1, 8, or 15, the cisplatin and gemcitabine doses were modified according to renal function. The gemcitabine dose was reduced to 75% and cisplatin dose to 50% if the serum creatinine was 120–150 μmol l^−1^. Serum creatinine >150 μmol l^−1^ led to treatment delay. Grade 3 mucosal or skin toxicity resulted in a dose reduction of 50% for gemcitabine until the toxicity abated. No dose adjustments were allowed for nausea and vomiting.

### Tumour assessment

Clinical history and examination, chest X-ray, vital capacity (FVC) and FEV_1_ were required prior to study entry and on day 1 of each cycle. A CT scan of the thorax and abdomen was performed prior to study entry, and a further CT scan of the thorax was performed prior to day 1 of the second, fourth, and sixth cycles and again at the end of the sixth cycle. Thereafter, CT scans were performed at 8 weekly intervals until disease progression. QOL was assessed using the EORTC QLQ-C30 questionnaire (Aaronson *et al*, 1993) supplemented by the lung cancer module QLQ-LC13, prior to study entry and on day 1 of cycles 2, 4, and 6, and 8 weekly thereafter until disease progression.

Tumour measurements were performed on transverse cuts on thoracic CT scans at three separate anatomically reproducible levels on the study entry CT scan and at the same levels on subsequent scans. Where possible, bidimensional lesions were measured. If there were no bidimensionally measurable lesions, unidimensional measurements of pleural tumour thickness were performed. Bidimensionally measurable lesions were measured using the longest dimension and the length perpendicular to the longest measurement. For unidimensionally measurable lesions, thickness of pleural tumour was measured at two separate sites on each level and the measurements summated to produce a total measurement. Palpable masses were measured clinically on day 1 of each cycle as for bidimensionally measurable lesions. A pleural effusion was not considered a measurable lesion.

### Tumour response

Tumour response was defined as: (1) complete response: disappearance of all known disease, determined by two observations not less than 4 weeks apart, (2) partial response: a 50% or greater decrease in the sum of the products of perpendicular diameters of bidimensionally measured lesions on two occasions not less than 4 weeks apart, or greater than or equal to a 30% decrease in the sum of linear tumour measurements on two observations not less than 4 weeks apart, (3) no change: a decrease in bidimensional tumour area of less than 50% or an increase of less than 25%; or a decrease in the sum of unidimensional measurements of less than 30% or an increase of less than 25%; provided no new lesions have appeared, (4) progressive disease: a 25% or greater increase in the size of the tumour being measured (unidimensional or bidimensional) or the appearance of new lesions.

Where response at sites measured bidimensionally differed from that at sites measured unidimensionally the overall patient response was assessed by the audit group. The sites with dominant tumour bulk were favoured.

### Assessment of endpoints

Patients who died of mesothelioma in whom there was no change or a response were regarded as having disease progression at the time of death. Time to disease progression was measured from the date of first chemotherapy dose to the first date of progression or death. Duration of response was measured from the first date of partial response to the first date of progression or death. Time to treatment failure was measured from day 1 of the first cycle of chemotherapy until the first of either disease progression, death in the absence of disease progression, or premature discontinuation of the protocol therapy. Survival was defined as the time from day 1 of the first cycle of chemotherapy until death.

Survival from the date of first diagnosis was also recorded. Treatment toxicity was recorded according to the National Cancer Institute (NCI) Common Toxicity Criteria, version 2.0.

### Treatment discontinuation

Treatment was discontinued on disease progression, following unacceptable toxicity in the opinion of the investigator, or on patient refusal to continue.

### Pathology and radiology review

Representative histological and cytological slides from each patient were centrally reviewed by one pathologist at Sir Charles Gairdner Hospital and a panel of immunohistochemical markers was performed on each case to ensure uniformity of diagnosis and histological subtyping. Initial CT scans of all patients were reviewed by a radiologist at Sir Charles Gairdner Hospital to ensure uniformity of staging according to UICC TNM criteria (Hermanek and Sobin, 1992). All CT scans were reviewed and measured for response centrally at Sir Charles Gairdner Hospital, and CT scans of patients considered responders were further audited by a panel of clinicians. If assessment of response or time of progression differed between the peripheral institution and central review at Sir Charles Gairdner Hospital, CT scans were also audited by a panel of clinicians who had final arbitration over response category.

### Statistical considerations

The study was designed to use the one-sample multiple testing procedure of Fleming (1982). The aim was to recruit a minimum of 15 patients initially, with a maximum of 35 patients in stages of 15, 10, and 10 patients. Analysis of the interim results was performed after each stage when patients became fully assessable. This allowed for early termination of the study if it became evident that the true rate of response (complete plus partial) was less than 20% or greater than 40%. Statistical analysis was conducted using the SPSS for Windows statistical package. Survival data were analysed with Kaplan–Meier survival curves. Quality of life data and lung function tests were not normally distributed and were analysed using the non-parametric Mann–Whitney *U* test. Differences were considered significant when the *P* value was less than 0.05.

## RESULTS

### Patient characteristics

Fifty-five patients were entered in the study, of whom 53 were eligible. The characteristics of the 53 eligible patients are listed in Table 1

**Table 1:**
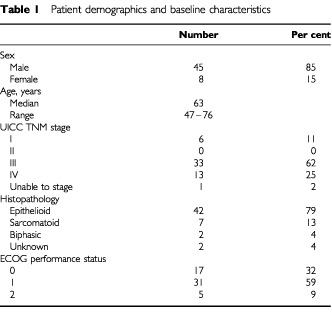
Patient demographics and baseline characteristics

. One patient with peritoneal mesothelioma without pleural disease was deemed ineligible, and another patient was found to have adenocarcinoma on pathology review and at subsequent autopsy. The 53 eligible patients were included in the analysis. As all patients were required to have measurable disease, the majority of patients had advanced disease at study entry with 46 (87%) having UICC TNM stage III or IV disease. Forty-eight patients (91%) had Eastern Cooperative Oncology Group (ECOG) performance status of 0 or 1. The majority of patients (79%) had epithelioid tumours. Three patients (6%) were diagnosed with malignant mesothelioma on cytology alone. One patient was judged to have non-measurable disease following central radiology review, as it was difficult to distinguish pleural fluid from solid lesions. This patient is not included in assessment of response, and is included as a ‘non-responder’ in analysis of QOL and pulmonary function tests. Nine (17%) patients had measurable subcutaneous lesions and 25 (48%) patients had at least one intrathoracic bidimensionally measurable lesion that was used in assessment of response.

### Treatment delivered

Nineteen patients completed the scheduled six cycles of treatment. The median number of cycles administered per patient was four. The relative dose intensity of cisplatin delivered was high (median, 100%; mean, 97%), however this was lower for gemcitabine (median, 72%; mean, 75%). Out of 227 planned day 1 treatments, 28 were delivered with a dose reduction. Out of 223 planned day 8 treatments, 39 were dose-reduced and 13 omitted. On day 15, 36 out of 219 planned treatments were dose-reduced and 90 omitted. The mean relative dose intensity delivered to responders (71%) was lower than that for patients with stable disease (78%) or progressive disease (73%) as best response. This reflects cumulative myelotoxicity as responders received more cycles of treatment than non-responders (mean 4.9 cycles *vs* 4.1 cycles).

### Response to treatment

**Figure 1:**
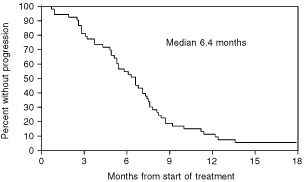
Time to progression (all patients) from start of treatment.

**Figure 2:**
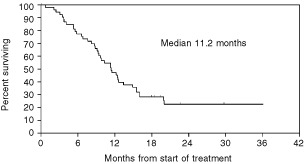
Overall survival from start of treatment.

No patient had a complete response. Seventeen patients (33%) had a partial response, and 31 patients (60%) had stable disease as their best response to treatment. Four patients (7%) had progressive disease, with no period of stabilisation. The overall response rate was 33% (95% CI 20–46%). The median duration of response was 5.4 months (range, 2.8–17.3 months) for those 17 patients achieving a partial response. The median time to progression was 6.4 months (Figure 1

). The median survival from start of treatment was 11.2 months (Figure 2

), and the median survival from diagnosis of malignant mesothelioma was 17.3 months for all patients. Responding patients had a median survival from start of treatment of 12.2 months, as compared with 9.1 months for non-responders. This difference was not significant. A partial response was observed in 13 out of 42 patients with epithelioid tumours (31%), in three out of seven patients with sarcomatoid tumours (43%), and in one of two patients with biphasic tumours. Out of five patients with ECOG performance status 2 at study entry, three had progressive disease as their best response, and only one attained a partial response.

### Toxicity

The major toxicities recorded were haematological, with 49% of patients experiencing grade 3 or 4 thrombocytopenia and 56% grade 3 or 4 neutropenia during treatment (Table 2

**Table 2:**
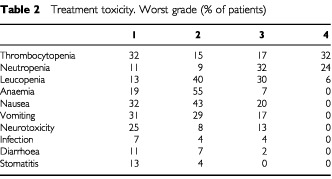
Treatment toxicity. Worst grade (% of patients)

). Infection however was uncommon, with only two patients (4%) developing febrile neutropenia during the course of treatment. There were no treatment-related deaths. Grade 1 and 2 anaemia was common, experienced by 74% of patients, but only 7% had grade 3 anaemia. Twenty-one patients required blood transfusion in 31 cycles, and 13 patients required platelet transfusion in 17 cycles. Nausea and vomiting were the major symptomatic toxicities, with 75 and 60% of patients respectively describing grade 1 or 2 nausea and vomiting, and 20 and 17% describing grade 3 nausea and vomiting. Thirteen percent of patients had grade 3 neurotoxicity, predominantly ototoxicity.

### Pulmonary function tests and quality of life

**Figure 3:**
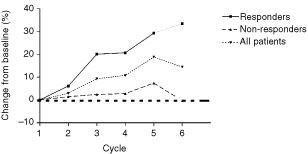
Percentage change in FVC from baseline over six cycles of chemotherapy. There was a significant difference between responding and non-responding patients during chemotherapy (*P*=0.002).

There was no significant change in FVC from baseline in the whole group (Figure 3

). There was a significant difference between responding (PR) and non-responding (SD and PD) patients (*P*=0.002) with an improvement in FVC in patients attaining partial response. FVC did not differ significantly from baseline in non-responding patients.

**Figure 4:**
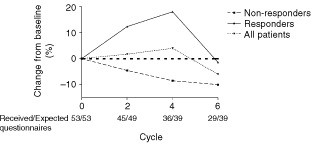
Global health status over six cycles of chemotherapy. Responding patients had a significantly improved global quality of life during treatment as compared with non-responders (*P*=0.008).

Among responding patients there was a significant improvement in the EORTC-QLQ-C30 global QOL scale (*P*=0.006) compared to non-responding patients over six cycles of chemotherapy (Figure 4

). This difference did not persist in questionnaires completed following the cessation of chemotherapy. In the patient group as a whole, there was no significant change from baseline in global QOL, nor in the physical, role, emotional, cognitive or social function scales during chemotherapy. There was no significant difference in the cognitive, role or social function scales between responders and non-responders. Responders showed improved physical function scores (*P*=0.01) as compared with non-responders, and non-responders showed improved emotional function scores (*P*=0.04) as compared with responders. In the EORTC-QLQ-C30 symptom scales, symptoms that improved significantly were appetite (at cycle six), diarrhoea, nausea and vomiting, and insomnia. There was no significant change in scores for pain, dyspnoea, constipation or financial problems. Fatigue was the only symptom scale which worsened from baseline in the group as a whole (*P*<0.005). There were no differences between responders and non-responders for any of the QLQ-C30 symptom scales. Only small numbers of patients reported pulmonary symptoms other than dyspnoea, cough and chest pain. As assessed by the QLQ-LC13 in the group as a whole, cough and chest pain scores decreased significantly from baseline at each time point. Dyspnoea remained generally stable but increased significantly at cycle six.

## DISCUSSION

This multicentre study confirms the efficacy of this 28-day regimen of cisplatin and gemcitabine in the treatment of pleural malignant mesothelioma, with an overall response rate of 33% accompanied by improved global QOL and FVC among responding patients. This suggests that the regimen has greater efficacy in this disease than most other reported single agents or combinations, as no drugs have consistently induced a response greater than 20% (Ong and Vogelzang 1996).

Neither cisplatin with a response rate of 13–14% (Mintzer *et al*, 1985; Zidar *et al*, 1988), nor gemcitabine (response rate 0–31%) (Bischoff *et al*, 1998; van Meerbeeck *et al*, 1999; Kindler *et al*, 2001) have shown great promise as single agents in the treatment of this disease. The additive or synergistic interaction of the two drugs documented *in vitro* (Peters *et al*, 1995) and in animal models (Peters *et al*, 1996) may underlie the useful clinical effects demonstrated in this study. Other combinations involving cisplatin (Thodtmann *et al*, 1999), or oxaliplatin (Fizazi *et al*, 2000) with other antimetabolites have shown promise in single studies and confirmatory studies with functional and QOL end points are awaited.

There is only one reported randomised phase III trial of chemotherapy in this disease. In a subset analysis which excluded patients with poor prognosis there was a survival advantage for patients receiving onconase over doxorubicin (11.3 *vs* 9.1 months) (Vogelzang *et al*, 2000).

The median survival of 11.2 months from start of treatment we observed is encouraging in a setting of advanced, measurable disease. When first seen, a number of patients had non-measurable disease and were not eligible for treatment. They became eligible weeks to months later as disease progressed. The inclusion requirement of measurable disease together with a life expectancy of more than 12 weeks may have selected for a group of patients with more indolent disease, which may have influenced survival from start of treatment and the unusually long median survival from diagnosis of 17.3 months. Survival is generally reported as 8–12 months. The question of whether this regimen improves survival can only be answered with a randomised controlled trial.

Patients with an ECOG performance status (PS) of 2 at study entry comprised the majority (75%) of those who had progressive disease as best response. Three out of five patients with PS 2 showed early progression, whilst one of 47 patients with PS 0–1 showed early progression. This is similar to the reported experience in non-small cell lung cancer, and patients with PS 2 are now excluded from many trials in this disease (Frasci *et al*, 2000; Schiller *et al*, 2000). Patients with pleural malignant mesothelioma who have a poor performance status may similarly derive less benefit from chemotherapy.

Our previous study suggested that patients with epithelioid tumours may have been more likely to respond to this combination. Sixty-nine per cent of patients with epithelioid tumours responded as compared with only 14% with biphasic tumours. Although the numbers of patients with biphasic and sarcomatoid tumours in this multi-center study were too small to perform a sub-group analysis, the response rate of 31% amongst those with epithelioid tumours and 43% amongst those with sarcomatoid tumours does not reinforce our previous observation.

Delivery of the full, scheduled dose intensity of gemcitabine was hampered by haematological toxicity, with dose reduction or omission commonly required at day 15, particularly in patients receiving more cycles of treatment. Dose omission was most commonly due to thrombocytopenia, while neutropenia was a frequent cause of dose reduction. Despite the common occurrence of severe neutropenia, there were only two reported incidents of febrile neutropenia. There were no deaths due to infection.

When our original pilot study of cisplatin and gemcitabine was initiated in 1995, this 28 day cycle with cisplatin given day 1 was the most well described schedule of delivery of this drug combination. This trial aimed to confirm the efficacy of the regimen in a multicentre setting, and thus we did not alter our established schedule in this second trial. Alternative schedules have been described for a variety of tumours. Some investigators have given cisplatin on days 2 or 15 of a 28 day cycle (Abratt *et al*, 1997; Crino *et al*, 1997), or split the cisplatin dose for days 1, 8 and 15 (Shepherd *et al*, 1997, 2000). Others have shortened the cycle to 21 days, administering cisplatin on day 1 (Rosell *et al*, 1998) or days 1 and 8 (Nagourney *et al*, 2000). Comparisons of toxicity between trials suffer from differing patient groups and definitions of haematological toxicity. There is, however, some consensus that schedules using cisplatin 100 mg m^−2^ on day 1 of a 28 day cycle are less able to deliver the full dose of gemcitabine due to frequent dose omissions and reductions for haematological toxicity on day 15 (Abratt *et al*, 1998). One small randomised trial tends to confirm the better tolerance of day 15 *vs* day 2 cisplatin delivery in a 28 day cycle (Ricci *et al*, 2000). A 21 day schedule may allow the delivery of a greater relative dose intensity of gemcitabine. It may also be useful to split the dose of cisplatin in an effort to decrease nausea and vomiting. However, we cannot assume that other schedules will be equally efficacious against malignant mesothelioma without appropriate clinical trials. A different schedule of this combination in malignant mesothelioma has been trialed by another group and reported in abstract form: gemcitabine 1250 mg m^−2^ days 1 and 8 and cisplatin 80 mg m^−2^ day 1 were administered to 29 patients on a 21 day cycle (van Haarst *et al*, 2000). Only four out of 22 evaluable patients (18%) had a partial response. WHO response criteria were used to evaluate response in this study. These criteria are difficult to apply to malignant mesothelioma in view of the unique growth pattern of tumour, as a ‘rind’ around the pleural cavity with less prominent bidimensionally measurable lesions. In contrast, our studies of this combination have included unidimensional measurements and response criteria similar to the RECIST criteria (Therasse *et al*, 2000) in addition to bidimensional measurements when possible. Thus the response rates in these studies may not be directly comparable.

We are aware of quality of life analysis in only one other study of chemotherapy in this disease. Steele *et al* (2000) treated 29 patients with weekly single agent vinorelbine 30 mg m^−2^. Twenty-four per cent achieved a partial response of their disease with a median survival from start of treatment of 10.6 months, and from diagnosis of 13.8 months. Quality of life data were collected 6-weekly using the Rotterdam Symptom checklist. After the first 6 weeks of treatment, 48% showed an improvement in lung related symptoms, 41% an improvement in general physical symptoms, and 76% an improvement in psychological symptoms. Activity levels worsened in 52% of patients. These figures were similar in patients continuing to further cycles of chemotherapy, and improvements were recorded in patients with stable disease as well as responding patients. It is not possible to directly compare the results of QOL data between these two trials due to the differences in measurement tools used, however both trials indicate that some patients obtain symptomatic and QOL benefit which may occur in the absence of objective response.

Given that FVC was significantly improved in responders, and remained unchanged from baseline in non-responders it was surprising to find no improvement in dyspnoea as assessed by the QLQ-C30 and the QLQ-LC13 instruments. We can hypothesise either that improvement in FVC may not translate to an improvement in dyspnoea, or was too small to demonstrate an improvement. This seems unlikely, given that the mean increase in FVC for responders at cycle 5 was over 30%. Alternatively, the three questions that comprise the ‘dyspnoea’ domain in the QLQ-LC13 may not be a sensitive reflection of changes in dyspnoea in patients with malignant mesothelioma. The high incidence of grade 2 and 3 anaemia, which was cumulative with increasing cycles of chemotherapy, may also contribute to a lack of perceived improvement in dyspnoea, as may treatment-related fatigue. It is also clear that the QLQ-LC13 Lung Cancer module may not be as well adapted for the symptom profile of malignant mesothelioma as it is for lung cancer. Symptoms of haemoptysis, arm pain, and dysphagia were rarely reported in our patients. However, troublesome chest wall masses and anorexia are relatively common symptoms in malignant mesothelioma which were not well assessed by this questionnaire. In the absence of an untreated control group it is difficult to assess whether this palliative chemotherapy regimen is beneficial to QOL. Whilst we would expect considerable deterioration over 6 months in the natural history of this disease, most domains do not appear to have deteriorated in the patient group as a whole over this period of chemotherapy.

We have confirmed in a multicentre setting that cisplatin and gemcitabine in this dose and schedule produces objective responses in a third of treated patients. Response to treatment is accompanied by improved global QOL and improved respiratory function. This regimen provides a satisfactory palliative treatment for some patients with advanced pleural mesothelioma.
